# Epidemiological characteristics of *Plasmodium malariae* malaria in China: a malaria that should not be neglected post elimination

**DOI:** 10.1186/s40249-023-01156-2

**Published:** 2023-11-20

**Authors:** Li Zhang, Bo-Yu Yi, Shui-Sen Zhou, Zhi-Gui Xia, Jian-Hai Yin

**Affiliations:** National Institute of Parasitic Diseases, Chinese Center for Disease Control and Prevention (Chinese Center for Tropical Diseases Research), NHC Key Laboratory of Parasite and Vector Biology, WHO Collaborating Center for Tropical Diseases, National Center for International Research On Tropical Diseases, Shanghai, 200025 China

**Keywords:** *Plasmodium malariae*, Imported case, Recurrence, Induced case, Reestablishment, China

## Abstract

**Background:**

*Plasmodium malariae* was always neglected compared with *P. falciparum* and *P. vivax*. In the present study, we aimed to describe the epidemiology of reported cases infected with *P. malariae* in the past decade to raise awareness of the potential threat of this malaria parasite in China.

**Methods:**

Individual data of malaria cases infected with *P. malariae* reported in China in the past decade were collected via the China Information System for Disease Control and Prevention and Parasitic Diseases Information Reporting Management System, to explore their epidemiological characteristics. Pearson Chi-square tests or Fisher’s Exact Test was used in the statistical analysis.

**Results:**

From 2013 to 2022, a total of 581 *P. malariae* cases were reported in China, and mainly concentrated in 20–59 years old group (*P* < 0.001), and there was no significant trend in the number of cases reported per month. Moreover, four kinds of *P. malariae* cases were classified, including 567 imported cases from 41 countries in 8 regions and distributed in 27 provinces (autonomous regions, municipalities) in China, six indigenous cases in a small outbreak in Hainan, seven recurrent cases in Guangdong and Shanghai, and one induced case in Shanghai, respectively. In addition, only 379 cases (65.2%) were diagnosed as malaria on the first visit (*P* < 0.001), and 413 cases (71.1%) were further confirmed as *P. malariae* cases (*P* = 0.002). Meanwhile, most cases sought healthcare first in the health facilities at the county and prefectural levels, but only 76.7% (161/210) and 73.7% (146/198) cases were diagnosed as malaria, and the accuracy of confirmed diagnosis as malaria cases infected with *P. malariae* was only 77.2% (156/202) and 69.9% (167/239) in these health facilities respectively.

**Conclusions:**

Even though malaria cases infected with *P. malariae* didn’t account for a high proportion of reported malaria cases nationwide, the threat posed by widely distributed imported cases, a small number of indigenous cases, recurrent cases and induced case cannot be ignored in China. Therefore, it is necessary to raise awareness and improve the surveillance and response to the non-falciparum species such as *P. malariae*, and prevent the reestablishment of malaria transmission after elimination.

**Graphical Abstract:**

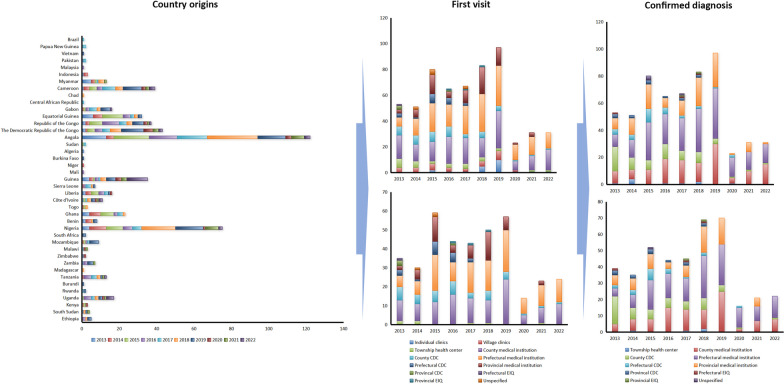

**Supplementary Information:**

The online version contains supplementary material available at 10.1186/s40249-023-01156-2.

## Background

Malaria is a public health threat, and considered as one of the “Big Three” infectious diseases [1]. Five major *Plasmodium* species including *Plasmodium falciparum*, *P. vivax*, *P. malariae*, *P. ovale* spp. (*P. ovale wallikeri*, *P. ovale curtisi*), and *P. knowlesi*, can infect human beings mainly through the bites of infected female *Anopheles* mosquitoes [2]. Furthermore, a few have also been reported to be transmitted through blood transfusions and maternal transmission with less frequent occurrences [3]. According to the World Malaria Report 2022, around 247 million malaria cases in 84 malaria endemic countries and 619,000 malaria-related deaths were estimated in 2021, and most of them were caused by *P. falciparum* and *P. vivax*, which especially occurred in the World Health Organization (WHO) African Region [4].

Compared to *P. falciparum* and *P. vivax* with particular scientific research interest, *P. ovale* spp. and *P. malariae* remain neglected due to their low prevalence with relatively lower disease severity [5–7]. Interestingly, it has been reported that *P. malariae* was frequently co-endemic with *P. falciparum* in sub-Saharan Africa, South America, Indonesia, Southeast Asia, and the western Pacific [3, 8]. Moreover, *P. malariae* is gaining more public health importance in the recent years due to the increasing prevalence in many parts of sub-Saharan Africa and South America [5], which is possible that the increasing use of sensitive malaria parasite detection techniques and the decline of *P. falciparum* prevalence after successful interventions in the endemic areas that may provide a favorable ecological niche for *P. malariae* expansion [7, 9–11]. In addition, *P. malariae* always causes chronic infections that are considered relatively benign, but may cause severe complications which may be fatal [12]. Meanwhile, chronic infections of *P. malariae* may last for decades and may recur long time after the initial exposure, which has raised concerns for elimination programmes, but the mechanisms for these recurrent infections remain unclear [13, 14].

In China, four human *Plasmodium* species (*P. falciparum*, *P. vivax*, *P. malariae*, *P. ovale*) have been prevalent in history, and *P. malariae* was relatively rare compared to *P. falciparum* and *P. vivax,* and diffusely distributed in southern China [15]. Since 2010, several recurrent cases have been reported for many years [16–20], and there was a small outbreak of *P. malariae* infections in 2015 [21]. Furthermore, it is challenging to correctly diagnose malaria cases caused by the relatively rare *Plasmodium* species such as *P. malariae*, *P. ovale* spp. and *P. knowlesi* [22], although there are detailed diagnosis requirements according to Diagnostic Criteria for Malaria (WS259-2006) [23] and the criteria on Diagnosis of Malaria (WS259-2015) [24] in China. In addition, malaria module of the Parasitic Diseases Information Reporting Management System has been fully operating since 2013 to push forward the national malaria elimination programme in China. Therefore, understanding the epidemiology of *P. malariae* cases is essential for the prevention of reestablishment of malaria transmission in China.

In the present study, the epidemiological characteristics of malaria cases infected with *P. malariae* alone reported in China in the past decade (2013–2022) are analyzed, which is intended to raise awareness of the potential threat of this malaria parasite. At the same time, this study will provide evidence to effectively address the corresponding challenges caused by *P. malariae* in the post-elimination phase, and ultimately contribute to the prevention of malaria re-establishment in China.

## Methods

### Data source and collection

Data on malaria cases infected with *P. malariae* alone reported in Chinese mainland (not include Hong Kong, Macao, and Taiwan) from January 1, 2013, to December 31, 2022, were extracted from the China Information System for Disease Control and Prevention and Parasitic Diseases Information Reporting Management System. Variables included case demographic information (age, gender), onset symptom, reporting time, diagnosis results of the first visit, confirmation and verification, health facilities for diagnosis, country origins of imported malaria cases, and geographical distribution in China.

All the reported cases were diagnosed and confirmed by microscopy, rapid diagnostic tests (RDT), or polymerase chain reaction (PCR) in the malaria diagnostic laboratory network, according to Diagnostic criteria for malaria (WS259-2006) [23] and the criteria on Diagnosis of Malaria (WS259-2015) in China [24]. And the procedures of malaria microscopy and RDT were basically in line with the manuals recommended by the WHO [25–27]. Moreover, microscopy is mainly used in the secondary medical institutions (hospitals or institutions for disease control and prevention) and above, and RDT is mainly used in medical facilities at township and village level (township hospitals, community health service centers, and village clinics at the border areas in Yunnan). In addition, PCR is always used in national and provincial reference laboratories for malaria diagnosis, but there are no uniform PCR methods. Furthermore, all the *Plasmodium*-positive samples are required to be verified by the provincial reference laboratories using PCR.

### Case definition

First, imported malaria case refers to the malaria case who traveled to malaria-endemic areas or had the previous malaria infection in history outside of China before the onset of illness, with evidence of case epidemiological investigation related to importation. Moreover, the country of origin for these imported malaria cases was defined as (1) the imported cases had a travel history to the countries where malaria transmission occurs, and (2) the onset time for malaria was less than 1 month after returning to China from this country. Second, indigenous case refers to a case contracted in China locally with no evidence of importation and without evidence to be as an introduced case. Third, recurrent case refers to a malaria case attributed to the recurrence of asexual parasitemia after antimalarial treatment, due to incomplete clearance of asexual parasitemia of the same genotype(s) that caused the original illness. Fourth, induced case refers to a case the origin can be traced to a blood transfusion or other form of parenteral inoculation of the parasite but not to transmission by a natural mosquito-borne inoculation. And *P. malariae* cases were required to be treated regularly according to the national treatment guidelines for *P. malariae* cases (Chloroquine is preferred. When chloroquine is ineffective, piperaquine phosphate, or pyronaridine or artemisinin-based combination therapies may be used) [28].

### Statistical analysis

The data extracted from the database was entered into a new worksheet using WPS Office (Kingsoft Office, Beijing, China), then checked by two authors individually. Geographic distribution statistics were also done by WPS Office. Then the comparative analysis between categorical variables among different groups was conducted with Pearson Chi-square tests or Fisher’s Exact Test using IBM SPSS Statistics Version 26.0 (IBM Corp., Armonk, NY, USA). The level of significance was set at *P* < 0.05 (two-sided).

## Results

**Fig. 1:**
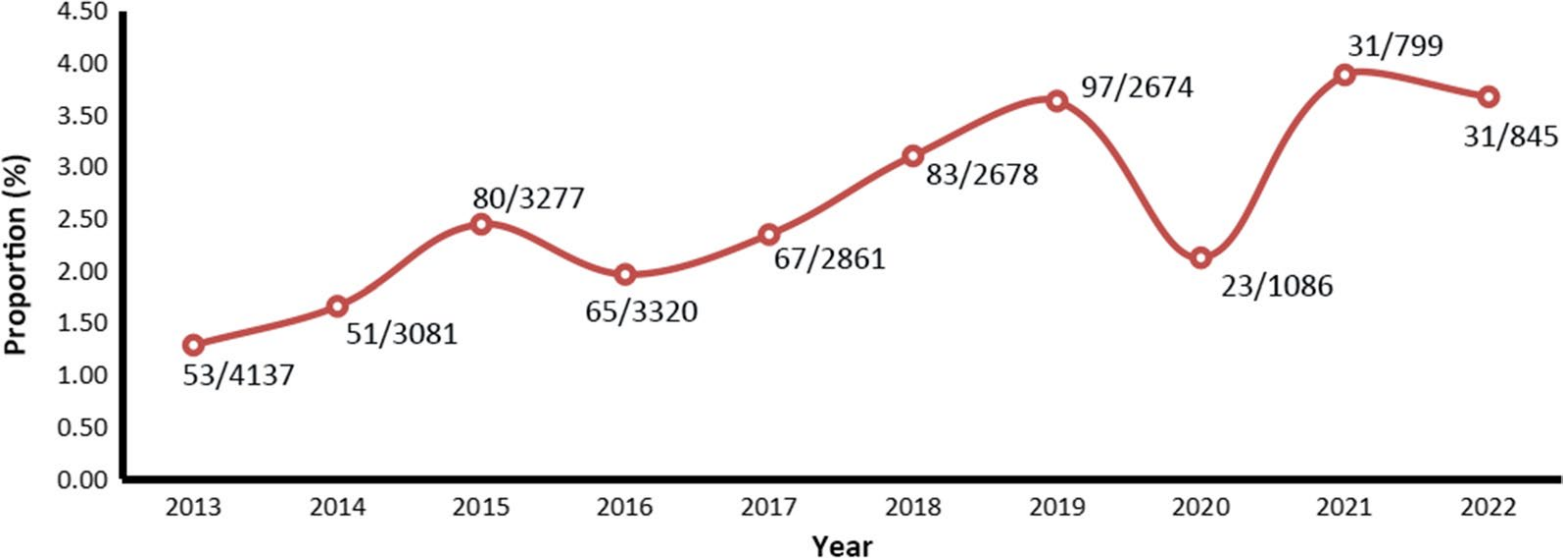
Proportion of malaria cases infected with *Plasmodium malariae* among the total malaria cases reported nationwide in China, 2013–2022

A total of 581 cases infected with *P. malariae* alone were reported from 2013 to 2022 in Chinese mainland with an overall slight increase, although it always represented a small percentage of the total cases reported nationally every year (1.3–3.9%) (Fig. 1), and 95.5% (555/581) of them are males (Table 1). The mean age of these cases was 40.6 ± 11.1 years old, ranging from 5 months old to 92 years old, and most of them (96.2%) were concentrated in 20–59 years old group (*P* < 0.001, Fisher’s Exact Test) (Table 1). And no asymptomatic cases and severe cases were reported.

**Table 1:** Age and gender distribution of reported cases infected with *Plasmodium malariae* from 2013 to 2022 in China

Age (Years)	Male		Female		Total	
	Number	Proportion (%)	Number	Proportion (%)	Number	Proportion (%)
< 10	2	0.3	2	0.3	4	0.7
10–19	1	0.2	1	0.2	2	0.3
20–29	83	14.3	7	1.2	90	15.5
30–39	168	28.9	4	0.7	172	29.6
40–49	181	31.2	4	0.7	185	31.8
50–59	111	19.1	1	0.2	112	19.3
60–69	7	1.2	1	0.2	8	1.4
≥ 70	2	0.3	6	1.0	8	1.4
Total	555	95.5	26	4.5	581	100.0

### Temporal distribution

**Fig. 2:**
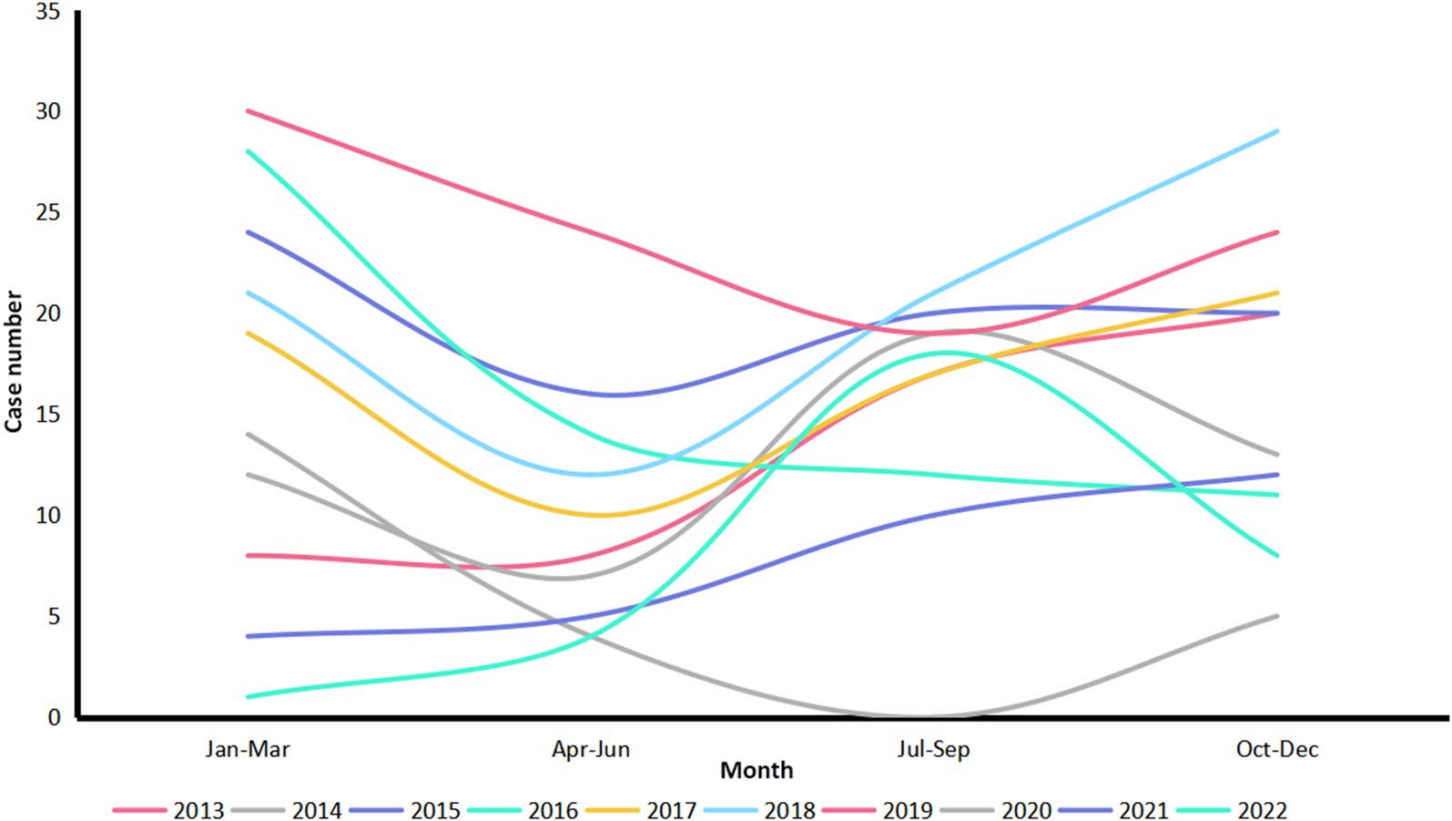
Quarterly distribution of the reported malaria cases infected with *Plasmodium malariae* reported in China, 2013–2022

Overall, there was no significant trend of cases reported by month, and no case was reported in April in 2015, July, August, September and November in 2020, March and May in 2021, and February, March and June in 2022, respectively. On a quarterly basis, relatively few cases were reported in the second quarter (April–June) (*χ*^2^ = 66.520, *df* = 27, *P* < 0.001) (Fig. 2).

### Case diagnosis, confirmation and treatment

**Fig. 3:**
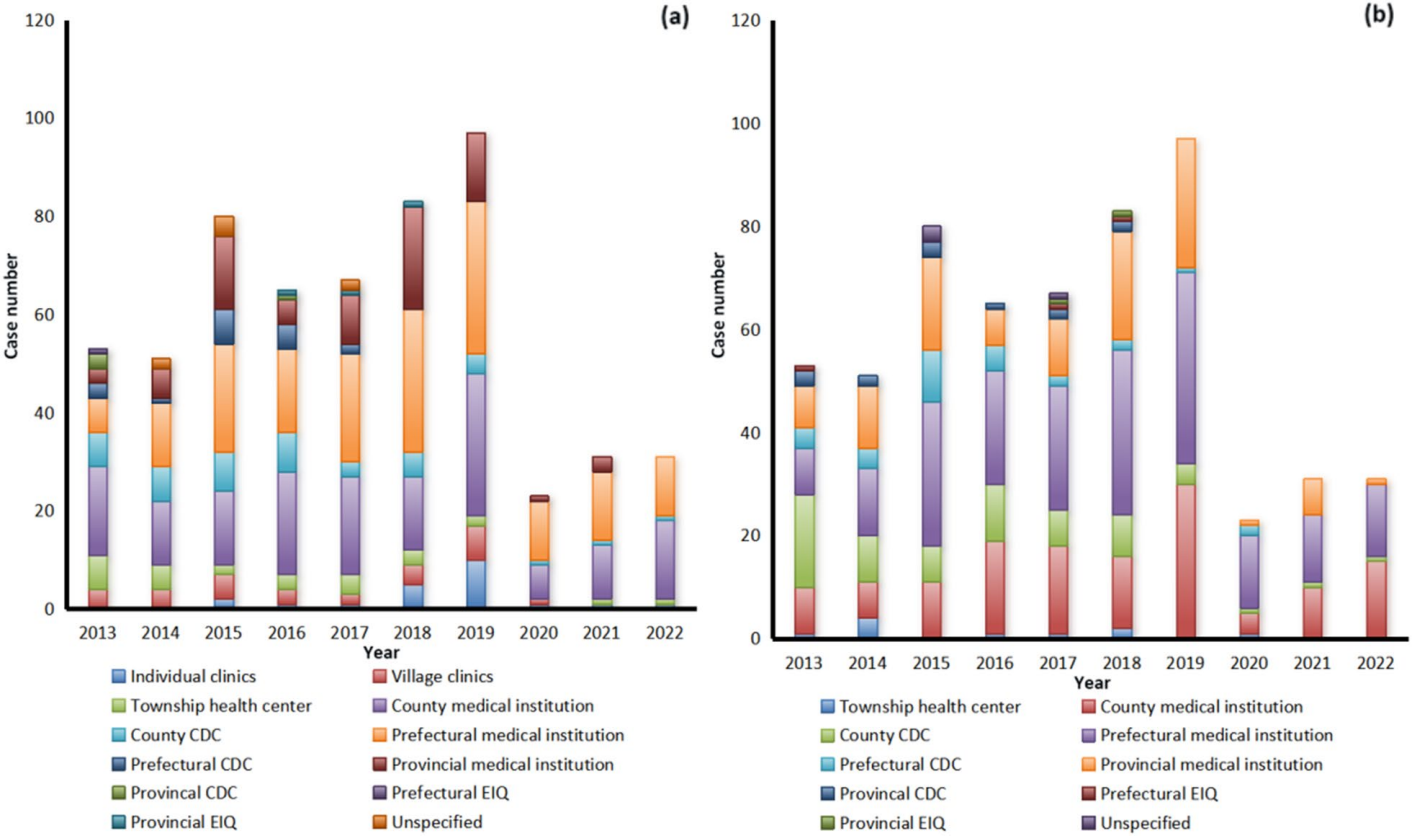
Distribution of reported cases infected with *Plasmodium malariae* at various health facilities nationwide in China, 2013–2022. **a** Case distribution in the first visit; **b** Case distribution when they were confirmed

**Fig. 4:**
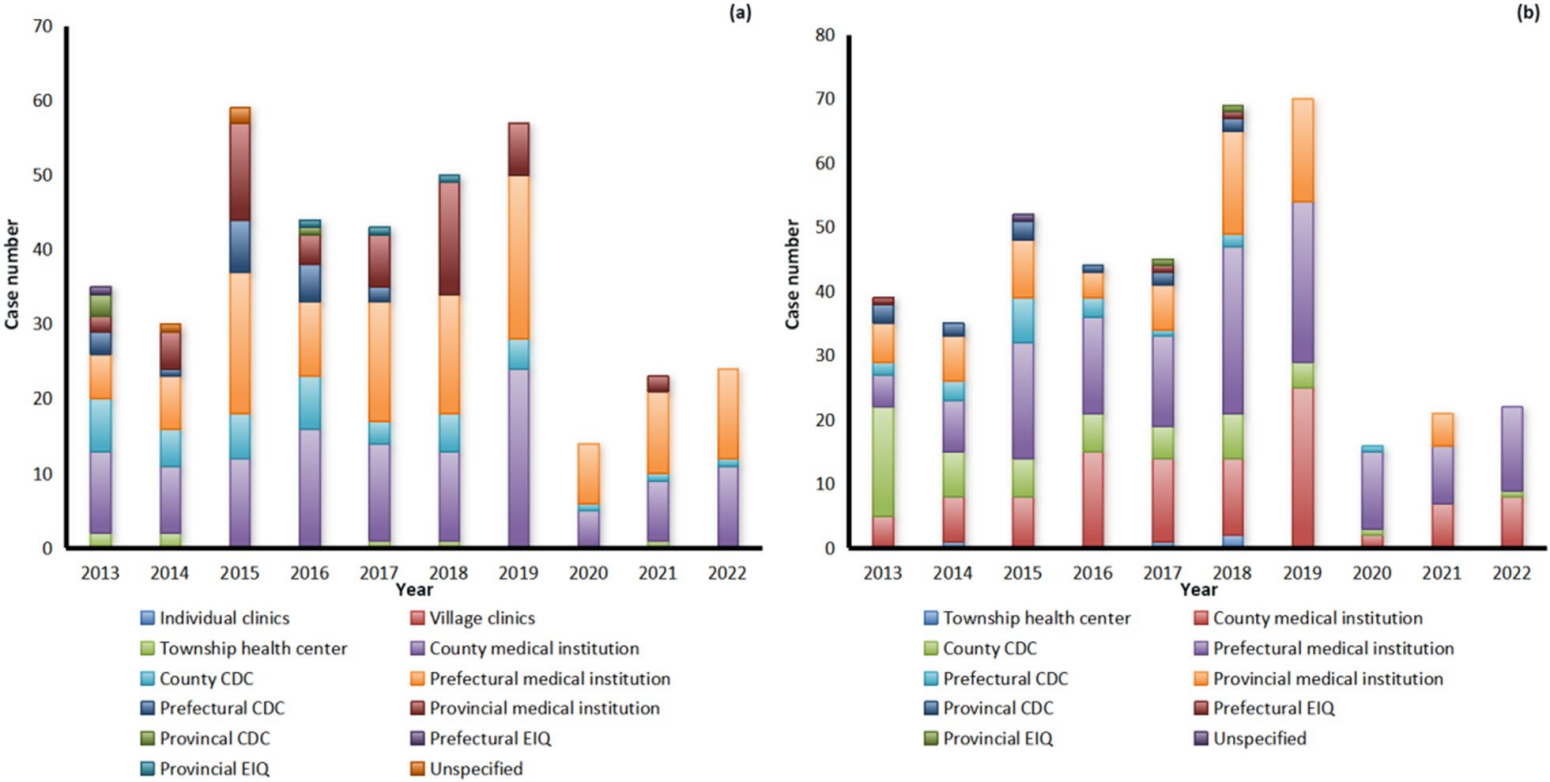
Distribution of malaria case infected with *Plasmodium malariae* diagnosed in the first visit and confirmation in various health facilities nationwide in China, 2013–2022. **a** Distribution of cases diagnosed as malaria cases in the first visit; **b** Distribution of cases confirmed as malaria cases infected with *P. malariae*

When the cases infected with *P. malariae* felt ill (fever, chill and headache), they first went to various medical and health institutions at different levels, including prefectural medical institutions (179), county medical institutions (165), provincial medical institutions (78), county center for disease control and prevention (CDCs) (45), village clinics (30), township health centers (28), individual clinics (22), prefectural CDCs (18), provincial CDCs (4), provincial entry-exit inspection and quarantine (EIQs) (3), prefectural EIQ (1), and no specific information on the institution of first visit was provided by the remaining 8 cases (Fig. 3a). Furthermore, they were mainly confirmed using microscopy by prefectural medical institutions (206), county medical institutions (135), provincial medical institutions (111), county CDCs (67), prefectural CDCs (30), provincial CDCs (13), township health centers (10), prefectural EIQ (3), provincial EIQs (2), and no specific information on the institution of confirmation was provided by the remaining 4 cases, respectively (Fig. 3b). However, only 379 cases (65.2%) were diagnosed as malaria on the first visit from 2013 to 2022 (*χ*^2^ = 133.243, *df* = 6, *P* < 0.001) (Table 2, Fig. 4a), and 413 cases (71.1%) were further confirmed as those infected with *P. malariae* (*P* = 0.002, Fisher’s Exact Test) (Table 2, Fig. 4b), and 153 cases (26.3%) were confirmed as those infected with other *Plasmodium* species, including *P. vivax* (70), *P. falciparum* (43), *P. ovale* spp. (28), untyping *Plasmodium* spp. (10), and mixed infections (2), respectively. Fortunately, all of the 581 cases infected with *P. malariae* were verified finally in the provincial reference laboratories for malaria diagnosis. Additionally, there were 403 and 175 patients who received inpatient and outpatient treatment, respectively, and three had unclear medical records. And most *P. malariae* cases were treated regularly according to the national treatment guidelines for *P. malariae* cases except for one case per year reported in 2016, 2017, 2018 and 2020.

**Table 2:** First diagnosis and confirmation of malaria case infected with *Plasmodium malariae* in various health facilities nationwide in China, 2013–2022

Health facilities	First visit (%)			Confirmed diagnosis (%)			
	Malaria	Other	Total	*P. malariae*	Other *Plasmodium* species	*Plasmodium* negative	Total
County	161 (27.7)	49 (8.4)	210 (36.1)	156 (26.9)	42 (7.2)	4 (0.7)	202 (34.8)
Prefectural	146 (25.1)	52 (9.0)	198 (34.1)	167 (28.7)	68 (11.7)	4 (0.7)	239 (41.1)
Provincial	62 (10.7)	23 (4.0)	85 (14.6)	85 (14.6)	37 (6.4)	4 (0.7)	126 (21.7)
Other	10 (1.7)	78 (13.4)	88 (15.1)	5 (0.9)	6 (1.0)	3 (0.5)	14 (2.4)
Total	379 (65.2)	202 (34.8)	581 (100.0)	413 (71.1)	153 (26.3)	15 (2.6)	581 (100.0)

### Case classification

Among all the reported cases infected with *P. malariae* alone from 2013 to 2022, most of them were imported cases (97.6%, 567/581), and 6 cases were indigenous cases, and 7 cases were recurrent cases, and one case was an induced case caused by blood transfusion (Additional file 1: Table S1).

### Imported case

**Fig. 5:**
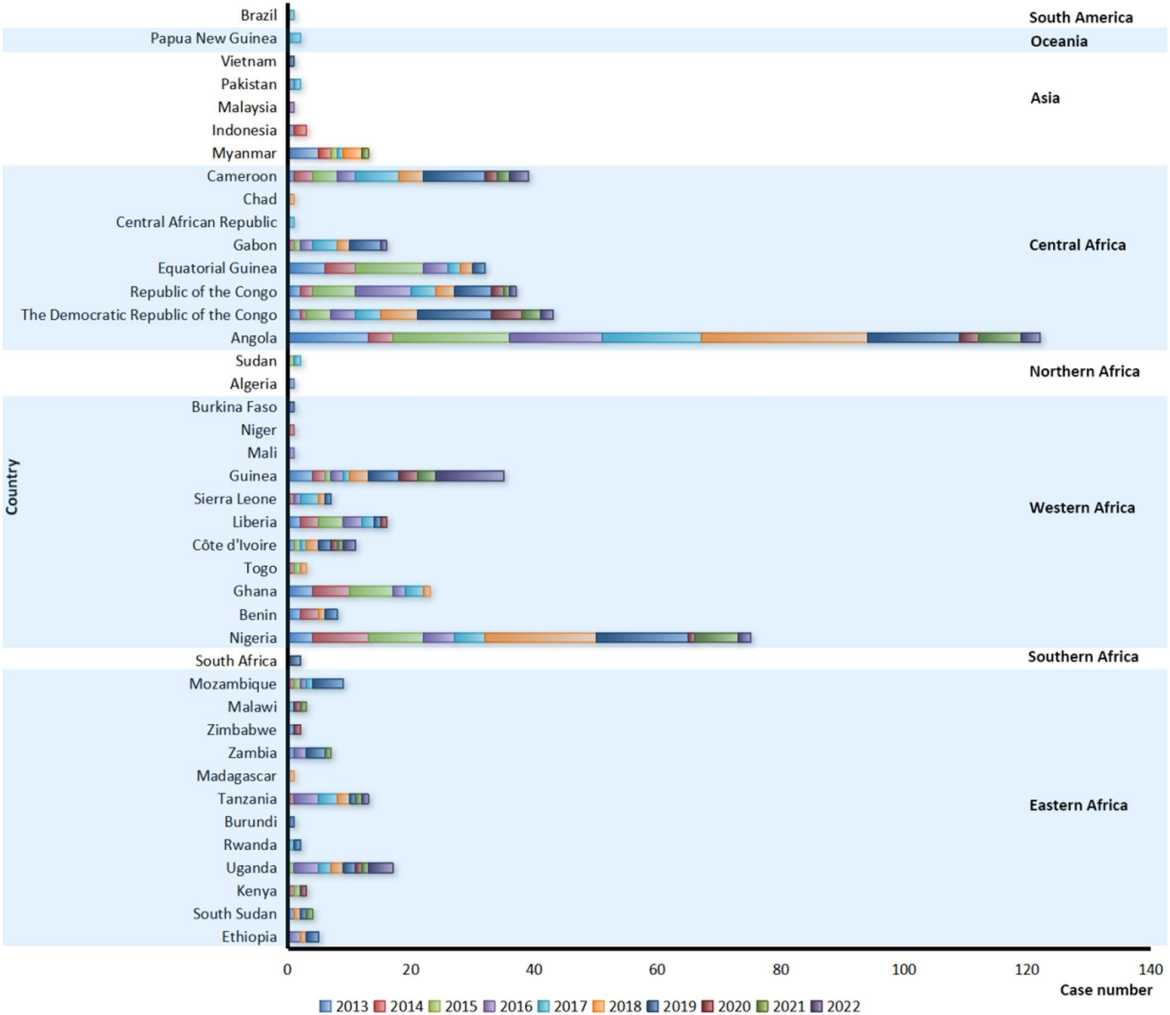
Country origins of the imported malaria cases infected with *Plasmodium malariae* reported in China, 2013–2022

**Fig. 6:**
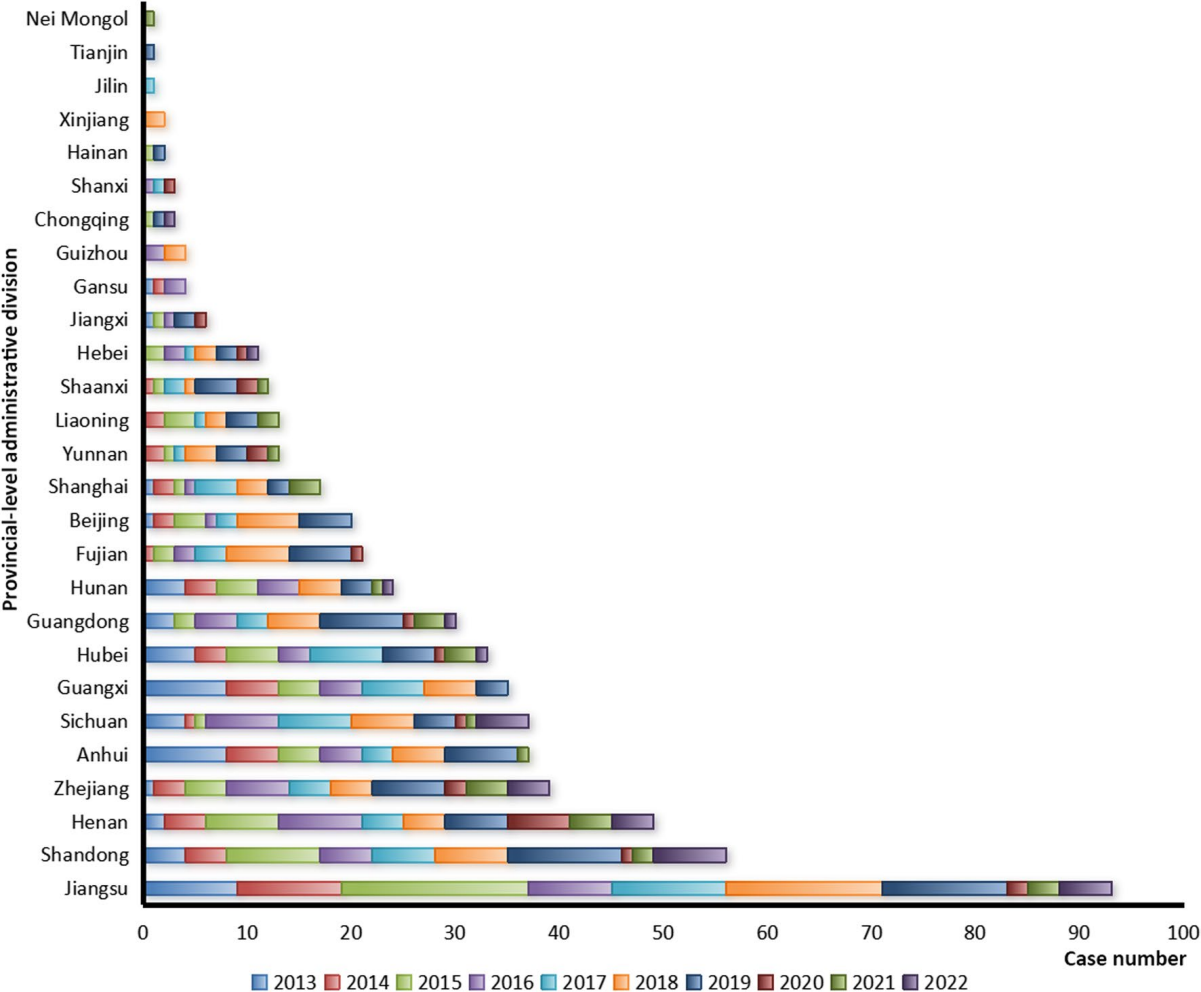
Provincial distribution of the imported malaria cases infected with *Plasmodium malariae* reported in China, 2013–2022

The imported cases (567) infected with *P. malariae* were from 41 countries in 8 regions (Fig. 5), and the highest number of cases came from Angola (122) in Central Africa (51.3%, 291/567) and Nigeria (75) in Western Africa (31.9%, 181/567) (Fig. 5). Meanwhile, these imported cases were distributed in 27 provinces (autonomous regions, municipalities) in China, and the largest number of reported cases were distributed in Jiangsu (93/567, 16.4%), Shandong (56/567, 9.9%), Henan (8.6%, 49/567), Zhejiang (6.9%, 39/567), Anhui (6.5%, 37/567) and Sichuan (6.5%, 37/567), respectively (Fig. 6, Additional file 2: Fig. S1).

### Indigenous case

A small outbreak of six indigenous malaria cases infected with *P. malariae* among forest goers from three villages (Baolong, Zhanan, Lixin) of Gaofeng Town in Sanya, Hainan Province, was reported from September to November 2015. All of them were male and aged from 19 to 40 years old, and were sequentially detected by blood smear microscopy and PCR in the passive case detection and active case detection.

### Recurrent case

A total of 7 cases of recurrent malaria cases infected with *P. malariae* have been reported in Shanghai and Guangdong since 2014, and all of them were without recent history of travel to a malaria-endemic areas. Among them, one case was reported in Jing'an District in Shanghai in 2014 (Male, 75 years old); Six cases were reported from Guangdong Province, namely Zhongshan in 2014 (Female, 70 years old), Guangzhou in 2018 (Male, 75 years old), 2019 (Female, 75 years old) and 2021 (Female, 76 years old), Qingyuan in 2020 (Female, 92 years old), and Jiangmen in 2022 (Female, 67 years old), respectively. And all the cases of *P. malariae* recurrence were mainly determined based on the following aspects: (1) *P. malariae* was characterized using the laboratory diagnosis; (2) A case recalled having suffered from malaria previously; (3) Recent infection with *P. malariae* was excluded based on evidences from case investigation and focus investigation including no recent travel history to malaria-endemic areas, and *P. malariae*-negative results of malaria screening in neighboring population.

### Induced case

An induced case (Male, 61 years old) infected with *P. malariae* due to the blood transfusion was confirmed by PCR and reported in Yangpu District in Shanghai in 2013.

## Discussion

From 2013 to 2022, a total of 581 cases infected with *P. malariae* alone were reported in China, and mainly concentrated in 20–59 years old, and no significant trend of cases reported by month. Meanwhile, four kinds of cases were classified, including 567 imported cases, 6 indigenous cases, 7 recurrent cases and one induced case.

Although a systematic review and meta-analysis of the reports from 2000 to 2020 revealed that no significant differences were found in the prevalence of *P. malariae* among adults, children and pregnant women [5], the majority of cases infected with *P. malariae* in China were the young and middle-aged male population due to most of them were migrant labor workers back from the malaria-endemic areas, which was consistent with the situation of all the imported cases nationwide [16–20, 29]. Moreover, *P. malariae* is still a neglected malaria parasite, even if it has a global distribution and frequent occurrence of co-infections with *P. falciparum* [6, 30], and the aggregate prevalence of *P. malariae* was estimated at 2.01% with significant differences among regions, such as the WHO Africa Region had the highest prevalence of 3.16%, the Region of the Americas revealed the second highest prevalence of 2.94%, and the Eastern Mediterranean Region showed the lowest prevalence of 0.06% [5]. Correspondingly, most imported cases infected with *P. malariae* reported in the past decade in China (95.9%, 544/567) were from African countries (Fig. 5). Historically, *P. malariae* was mainly distributed in the area south of 33°north latitude of China, and it was widely distributed in south of the Qinling Mountains, but it was scattered and was not the dominant species [15]. Furthermore, the species of *Anopheles* mosquitoes, such as *An. minimus*, *An. dirus*, *An. messeae*, *An. jeyporiensis*, *An. culicifacies*, *An. maculatus*, *An. saccharovi*, *An. sinensis*, etc., that have been documented to be infected with *P. malariae* worldwide [3], have also been recorded in China [15]. Therefore, given the fact that imported cases infected with *P. malariae* were widely distributed in 27 provinces (autonomous regions, municipalities) in China in the past decade, coupled with a small indigenous outbreak in Hainan particularly, it is very necessary to carry out systematic surveillance of malaria vector *Anopheles*, rather than only focusing on the four main malaria-transmitting *Anopheles* mosquitoes (*An. sinensis*, *An. minimus*, *An. dirus*, and *An. anthropophagus*) in China [31], so as to more accurately assess the risk of reestablishment of malaria transmission.

In the post elimination phase, prompt and precise diagnosis of malaria cases is even more vital for the prevention of malaria reestablishment. However, laboratory diagnostic capacity for malaria still faces challenges [32], especially in the county-level and prefectural health institutions with the largest number of malaria cases, with an overall detection rate of 75% and 90% accuracy in the first visit and confirmed diagnosis [22], respectively. For relatively rare species such as *P. malariae*, the diagnostic performance is even less satisfactory [22]. For example, the first diagnosis as malaria at the county and prefectural levels was 76.7% (161/210) and 73.7% (146/198), while the accuracy of confirmed diagnosis as malaria cases infected with *P. malariae* was only 77.2% (156/202) and 69.9% (167/239) respectively in the past decade (Table 2).

Regarding the small-scale outbreak of malaria cases infected with *P. malariae* in Hainan, the cause is still not clear, and there are several possibilities. The first is the secondary transmission caused by the unknown *P. malariae* infection through the *Anopheles* mosquitoes biting; Second, Sanya is an international tourist city, and there may be secondary transmission caused by imported cases; The third is that *P. malariae* is considered as a zoonotic malaria parasite [33], which can be transmitted from monkeys to humans through suitable *Anopheles* bites [34], and the patients in this outbreak had a history of uphill operations, and monkeys do live on the mountain, but the malaria parasite infection of monkeys is unknown and deserves further exploration. As far as is known, *P. malariae* blood stage can persist for extremely long periods, even for the life of the human host. As no dormant liver stages of hypnozoites have been reported for this species, and the mechanisms for these recurrent infections remain unclear [13, 14]. Interestingly, Guangdong Province has been reporting recurrent cases infected with *P. malariae* in the elderly in different places for many years [16–20, 35]. This requires further analysis of the characteristics of these cases in Guangdong Province, identifying appropriate key populations and strengthening case surveillance and malaria vector surveillance using more sensitive nucleic acid detection techniques. Additionally, for the induced case caused by blood transfusion, the blood was positive of *P. malariae* based on the retrospective testing, and the blood was donated by an international student from Côte d’Ivoire [36], thus it is recommended to strengthen malaria parasite screening for donated blood, especially for blood donors with a history of travel and residence in malaria-endemic areas, thus ensuring healthy blood for transfusion [37, 38].

There are some limitations in this study. First, no detailed data on diagnostic methods used in the first diagnosis and confirmed diagnosis were available. Second, although the Pearson chi-square test and Fisher’s exact tests are the most common methods for understanding tests of the association of categorical variables, there may be other better statistical methods to analyze the data from this study. Third, we only focused on the epidemiological characteristics of malaria cases infected with *P. malariae* in China in the past decade, rather than a complete picture of the burden caused by this malaria parasite.

## Conclusions

In China, the threat of *Plasmodium malariae* cannot be ignored, a large number of imported cases were widely distributed in areas with suitable vector *Anopheles* mosquitoes, and there were some recurrent cases (especially in Guangdong Province), a small outbreak during the elimination phase, and even an induced case of blood transfusion infection has been reported. At the same time, the detection capacity of *P. malariae*, especially in the county and prefectural health institutions that received the most patients, cannot fully meet the needs of timely detection of infectious sources. Therefore, it is necessary to raise awareness of the threat of the rare malaria parasite such as *P. malariae*, and a particular focus towards improved surveillance and response through strengthening the research and development of advanced diagnostic tools as well as the diagnosis competency in the health facilities should be stressed upon to prevent the reestablishment of malaria transmission post elimination in China.

### Supplementary Information

**Additional file 1. Table S1.** Classification of reported malaria cases infected with *Plasmodium malariae* in China, 2013–2022.

**Additional file 2. Figure S1.** Map of China, Map approval number GS(2019)1671.

## Data Availability

The datasets used and/or analyzed during the present study are available from the corresponding author upon reasonable request.

## Abbreviations

WHO: World Health Organization
RDT: Rapid diagnostic tests
PCR: Polymerase chain reaction
CDC: Center for disease control and prevention
EIQ: Entry-exit inspection and quarantine

## References

[1] Makam P, Matsa R (2021). “Big Three” infectious diseases: tuberculosis, malaria and HIV/AIDS. Curr Top Med Chem.

[2] WHO. Malaria. https://www.who.int/news-room/fact-sheets/detail/malaria. Accessed Jun 11, 2023.

[3] Collins WE, Jeffery GM (2007). *Plasmodium malariae*: parasite and disease. Clin Microbiol Rev.

[4] World malaria report 2022. Geneva: World Health Organization; 2022.

[5] Hawadak J, Dongang Nana RR, Singh V (2021). Global trend of *Plasmodium malariae* and *Plasmodium ovale* spp. malaria infections in the last two decades (2000–2020): a systematic review and meta-analysis. Parasit Vectors.

[6] Su X, Lane KD, Xia L, Sá JM, Wellems TE (2019). *Plasmodium* genomics and genetics: new insights into malaria pathogenesis, drug resistance, epidemiology, and evolution. Clin Microbiol Rev.

[7] Yman V, Wandell G, Mutemi DD, Miglar A, Asghar M, Hammar U (2019). Persistent transmission of *Plasmodium malariae* and *Plasmodium ovale* species in an area of declining *Plasmodium falciparum* transmission in eastern Tanzania. PLoS Negl Trop Dis.

[8] Mayxay M, Pukrittayakamee S, Newton PN, White NJ (2004). Mixed-species malaria infections in humans. Trends Parasitol.

[9] Mueller I, Zimmerman PA, Reeder JC (2007). *Plasmodium malariae* and *Plasmodium ovale*–the “bashful” malaria parasites. Trends Parasitol.

[10] Oriero EC, Amenga-Etego L, Ishengoma DS, Amambua-Ngwa A (2021). *Plasmodium malariae*, current knowledge and future research opportunities on a neglected malaria parasite species. Crit Rev Microbiol.

[11] Culleton R, Pain A, Snounou G (2023). *Plasmodium malariae*: the persisting mysteries of a persistent parasite. Trends Parasitol.

[12] Kotepui M, Kotepui KU, Milanez GD, Masangkay FR (2020). Global prevalence and mortality of severe *Plasmodium malariae* infection: a systematic review and meta-analysis. Malar J.

[13] Vinetz JM, Li J, McCutchan TF, Kaslow DC (1998). *Plasmodium malariae* infection in an asymptomatic 74-year-old Greek woman with splenomegaly. N Engl J Med.

[14] Hedelius R, Fletcher JJ, Glass WF, Susanti AI, Maguire JD (2011). Nephrotic syndrome and unrecognized *Plasmodium malariae* infection in a US Navy sailor 14 years after departing Nigeria. J Travel Med.

[15] Tang LH, Xu LQ, Chen YD. Parasitic Disease Control and Research in China. Beijing: Beijing Science and Technology Press. 2012; 75–82.

[16] Zhang L, Feng J, Zhang SS, Xia ZG, Zhou SS (2019). Epidemiological characteristics of malaria and the progress towards its elimination in China in 2018. Zhongguo Ji Sheng Chong Xue Yu Ji Sheng Chong Bing Za Zhi.

[17] Zhang L, Feng J, Xia ZG, Zhou SS (2020). Epidemiological characteristics of malaria and progress on its elimination in China in 2019. Zhongguo Ji Sheng Chong Xue Yu Ji Sheng Chong Bing Za Zhi.

[18] Zhang L, Feng J, Tu H, Yin JH, Xia ZG (2021). Malaria epidemiology in China in 2020. Zhongguo Ji Sheng Chong Xue Yu Ji Sheng Chong Bing Za Zhi.

[19] Zhang L, Yi BY, Xia ZG, Yin JH (2022). Epidemiological characteristics of malaria in China, 2021. Zhongguo Ji Sheng Chong Xue Yu Ji Sheng Chong Bing Za Zhi.

[20] Zhang L, Yi BY, Yin JH, Xia ZG (2023). Epidemiological characteristics of malaria in China, 2022. Zhongguo Ji Sheng Chong Xue Yu Ji Sheng Chong Bing Za Zhi.

[21] Zhang L, Feng J, Zhang SS, Xia ZG, Zhou SS (2016). Malaria situation in the People’s Republic of China in 2015. Zhongguo Ji Sheng Chong Xue Yu Ji Sheng Chong Bing Za Zhi.

[22] Yin J, Zhang L, Feng J, Zhou S, Xia Z (2020). Malaria diagnosis and verification—China, 2017–2018. China CDC Wkly.

[23] Ministry of Health, the People’s Republic of China. Diagnostic Criteria for Malaria (WS 259-2006). 2006.

[24] National Health and Planning of the People’s Republic of China. Diagnosis of malaria (WS 259-2015). 2015.

[25] Basic malaria microscopy-2nd edition. Geneva: World Health Organization; 2010.

[26] How to use a rapid diagnostic test (RDT): A guide for training at a village and clinic level (Modified for training in the use of the Generic *Pf* Test for falciparum malaria). Bethesda, MD, and Geneva: The USAID Quality Assurance Project (QAP), University Research Co., LLC, and the World Health Organization (WHO); 2009.

[27] How to use a rapid diagnostic test (RDT): A guide for training at a village and clinic level (Modified for training in the use of the Generic *Pf*-Pan Test for falciparum and non-falciparum malaria). Bethesda, MD, and Geneva: The USAID Quality Assurance Project (QAP), University Research Co., LLC, and the World Health Organization (WHO); 2009.

[28] National Health and Planning of the People’s Republic of China. Technical regulations for application of antimalarials (WS/T 485–2016). 2016.

[29] Yin JH, Zhang L, Yi BY, Zhou SS, Xia ZG (2023). Imported malaria from land bordering countries in China: a challenge in preventing the reestablishment of malaria transmission. Travel Med Infect Dis.

[30] Amoah LE, Donu D, Abuaku B, Ahorlu C, Arhinful D, Afari E (2019). Probing the composition of *Plasmodium* species contained in malaria infections in the Eastern region of Ghana. BMC Public Health.

[31] Bureau of Disease Control and Prevention, Ministry of Health. Manual on malaria control and prevention (3rd edition). Beijing: People’s Medical Publishing House. 2007; 47–85.

[32] Yin J, Li M, Yan H, Zhou S, Xia Z (2022). Laboratory diagnosis for malaria in the elimination phase in China: efforts and challenges. Front Med.

[33] Plenderleith LJ, Liu W, Li Y, Loy DE, Mollison E, Connell J (2022). Zoonotic origin of the human malaria parasite *Plasmodium malariae* from African apes. Nat Commun.

[34] Contacos PG, Collins WE (1969). *Plasmodium malariae*: transmission from monkey to man by mosquito bite. Science.

[35] Zhang L, Zhou SS, Feng J, Fang W, Xia ZG (2015). Malaria situation in the People’s Republic of China in 2014. Zhongguo Ji Sheng Chong Xue Yu Ji Sheng Chong Bing Za Zhi.

[36] Wang ZY, Zhang YG, Jiang L, Li M, Zhu M, Li C (2015). Laboratory analysis and diagnosis of one transfusion-transmitted quartan malaria case in Shanghai City. Zhongguo Xue Xi Chong Bing Fang Zhi Za Zhi..

[37] Batista-Dos-Santos SA, Freitas DRC, Raiol M, Cabral GF, Feio AC, Póvoa MM (2018). Strategy to improve malaria surveillance system preventing transfusion-transmitted malaria in blood banks using molecular diagnostic. Malar J.

[38] Ministry of Health, the People’s Republic of China. Whole blood and component donor selection requirements (GB18467-2011). http://www.nhc.gov.cn/wjw/s9493/201207/55286/files/1fafcf615e054334ace37019569951cf.PDF. Accessed 23 Aug 2023.

