# Supplementary material for: Epidemiological characteristics of Plasmodium malariae malaria in China: a malaria that should not be neglected post elimination
Source: Infect Dis Poverty. 2023 Nov 20;12:101. doi: 10.1186/s40249-023-01156-2 (PMC10658989; doi:10.1186/s40249-023-01156-2)
Supplement: Supplementary file 1 — Additional file 1. Table S1. Classification of reported malaria cases infected with Plasmodium malariae in China, 2013–2022. [file 40249_2023_1156_MOESM1_ESM.docx]

Additional file 1: Table S1 Classification of reported malaria cases infected with *Plasmodium malariae* in China, 2013-2022

| **Case classification** | **2013** | **2014** | **2015** | **2016** | **2017** | **2018** | **2019** | **2020** | **2021** | **2022** | **Total** |
| --- | --- | --- | --- | --- | --- | --- | --- | --- | --- | --- | --- |
| **Imported case** | 52 (9.0) | 49 (8.4) | 74 (12.7) | 65 (11.2) | 67 (11.5) | 82 (14.1) | 96 (16.5) | 22 (3.8) | 30 (5.2) | 30 (5.2) | 567 (97.6) |
| **Indigenous case** | 0 | 0 | 6 (1.0) | 0 | 0 | 0 | 0 | 0 | 0 | 0 | 6 (1.0) |
| **Recurrent case** | 0 | 2 (0.3) | 0 | 0 | 0 | 1 (0.2) | 1 (0.2) | 1 (0.2) | 1 (0.2) | 1 (0.2) | 7 (1.2) |
| **Induced case** | 1 (0.2) | 0 | 0 | 0 | 0 | 0 | 0 | 0 | 0 | 0 | 1 (0.2) |
| **Total** | 53 (9.1) | 51 (8.8) | 80 (13.8) | 65 (11.2) | 67 (11.5) | 83 (14.3) | 97 (16.7) | 23 (4.0) | 31 (5.3) | 31 (5.3) | 581 (100.0) |
