# Supplementary figures and images for: Epidemiological characteristics of Plasmodium malariae malaria in China: a malaria that should not be neglected post elimination
Source: Infect Dis Poverty. 2023 Nov 20;12:101. doi: 10.1186/s40249-023-01156-2 (PMC10658989; doi:10.1186/s40249-023-01156-2)

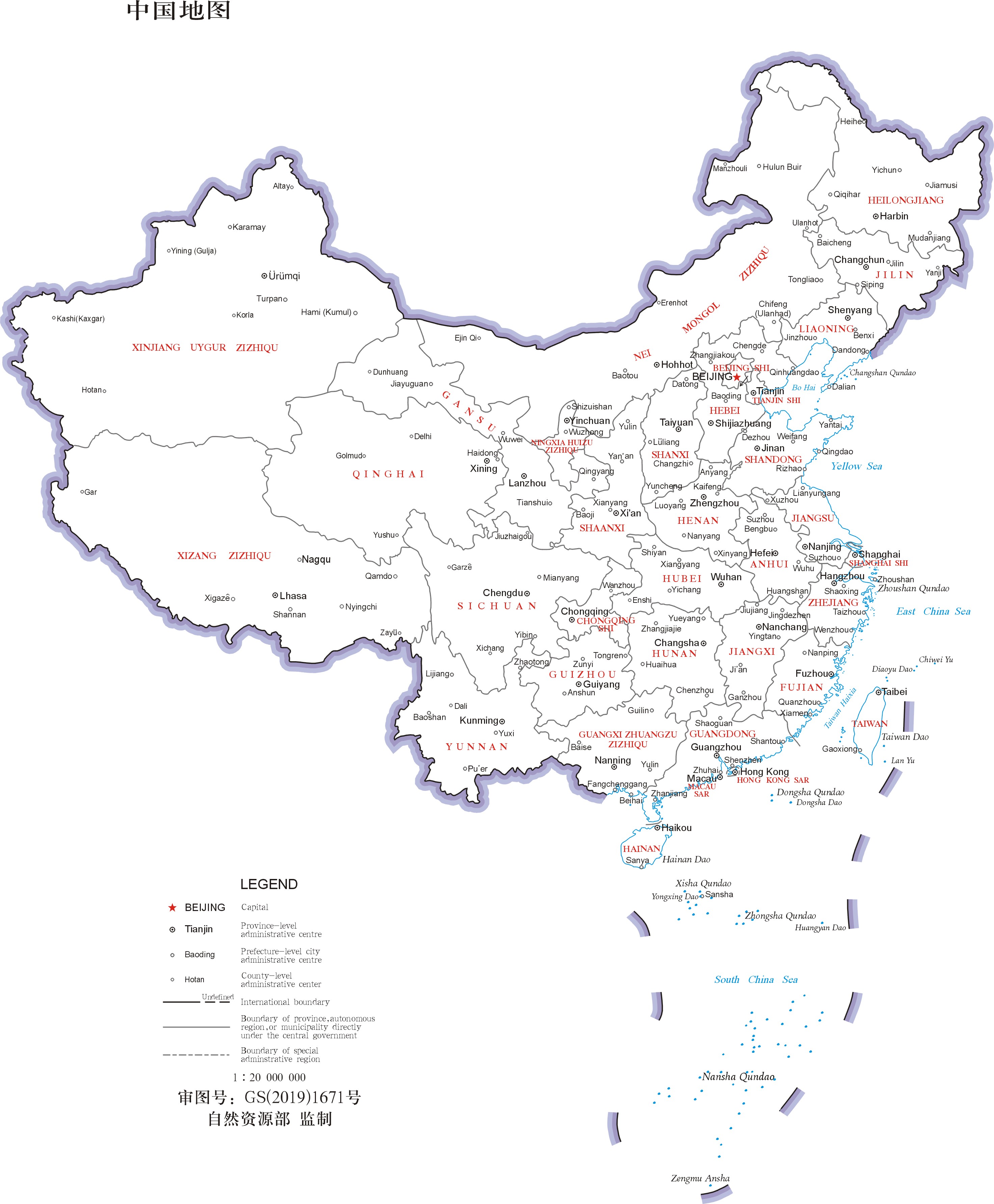

Supplement: Supplementary file 2 — Additional file 2. Figure S1. Map of China, Map approval number GS(2019)1671. [file 40249_2023_1156_MOESM2_ESM.jpg]
